# 908. Impact of Antimicrobial Stewardship Protocol Mandating Infectious Diseases Consultation Post 72 Hours of Meropenem Usage

**DOI:** 10.1093/ofid/ofac492.753

**Published:** 2022-12-15

**Authors:** Nhi Nguyen, Rachel S Britt, David Reynoso, Scott Ferren

**Affiliations:** The University of Texas Medical Branch, Las Vegas, Nevada; The University of Texas Medical Branch, Las Vegas, Nevada; The University of Texas Medical Branch, Las Vegas, Nevada; The University of Texas Medical Branch, Las Vegas, Nevada

## Abstract

**Background:**

Carbapenem use correlates with the development of carbapenem-resistant organisms, prompting our antimicrobial stewardship program (ASP) to develop a policy mandating infectious diseases (ID) consultation after 72 hours of meropenem use. Our objective was to evaluate the impact of this policy on meropenem utilization and associated clinical outcomes.

**Methods:**

This quasi-experimental, observational study evaluated the impact of the new ASP policy in adult patients across four campuses. Administered meropenem orders were retrieved retrospectively six months before (11/2020-4/2021) and after (6/2021-11/2021) policy implementation. The primary outcome was meropenem days of therapy per 1000 patient-days (DOTs). Secondary outcomes included DOTs of select broad-spectrum antimicrobials, 30-day all-cause mortality, hospital length of stay (LOS), and *Clostridioides difficile* (*C. difficile*) infection incidence. All outcomes were assessed in pre- and post-intervention periods.

**Results:**

There were 1493 and 1404 meropenem orders in the pre- and post-intervention periods, respectively. Pre-intervention group patients had slightly higher modified Charlson Comorbidity Index scores (2.1 vs 1.9, p=0.02). Pre-intervention group had more patients with penicillin allergies but less patients with sulfa allergies (p=0.007 and p=0.03, respectively). The most common meropenem indications were bloodstream, respiratory, abdominal, and urinary tract infections. The incidence of extended-spectrum beta-lactamase (ESBL)-producing Enterobacterales and cefepime-resistant *Pseudomonas* spp. was similar in both groups (p=0.7 and p=0.1, respectively). ID consultation increased after policy implementation (44.1% vs 51.7%, p=0.001). Meropenem DOTs decreased significantly after intervention (50.3 vs 35.5, p=0.0003). We observed increases in ceftriaxone and cefepime DOTs (94 vs 103.8, p=0.006 and 39 vs 58.5, p=0.0005, respectively). An increase in *C. difficile* incidence was seen. Hospital LOS was similar pre- and post-intervention (mean 18.5 days, p=0.9).

**Conclusion:**

The ASP policy mandating ID consultation after 72 hours of meropenem use helped decrease meropenem DOT and encouraged use of antimicrobial agents with narrower spectrum.

**Disclosures:**

**All Authors**: No reported disclosures.

